# Health care utilization and its association with sociodemographic factors among slum‐dwellers with type 2 diabetes in Tabriz, Iran: A cross‐sectional study

**DOI:** 10.1002/hsr2.1272

**Published:** 2023-05-25

**Authors:** Fawzieh Ghammari, Habib Jalilian, Rahim Khodayari‐zarnaq, Masumeh Gholizadeh

**Affiliations:** ^1^ Department of Health Policy and Management, School of Management and Medical Informatics Tabriz University of Medical Sciences Tabriz Iran; ^2^ Department of Health Services Management, School of Health Ahvaz Jundishapur University of Medical Sciences Ahvaz Iran

**Keywords:** diabetes mellitus, health care utilization, poverty areas, Type 2

## Abstract

**Background and Aims:**

Slums are known as growing underprivileged areas. One of the health adverse effects of slum‐dwelling is health care underutilization. Management of type 2 diabetes mellitus (T2DM) requires an appropriate utilization. This study aimed to investigate the extent of health care utilization among slum‐dwellers with T2DM in Tabriz, Iran, in 2022.

**Methods:**

We conducted a cross‐sectional study on 400 patients with T2DM living in slum areas of Tabriz, Iran. Sampling was conducted using a systematic random sampling method. A researcher‐made questionnaire was used for data collection. To develop the questionnaire, we used Iran's Package of Essential Noncommunicable (IraPEN) diseases, in which potential needs and essential health care for patients with diabetes and the appropriate time intervals for use are specified. Data were analyzed using SPSS version 22.

**Results:**

Although 49.8% of patients needed outpatient services, only 38.3% were referred to health centers and utilized health services. The results of the binary logistic regression model showed that women (OR = 1.871, CI 1.170–2.993), those with higher income levels (OR = 1.984, CI 1.105–3.562), and those with diabetes complications (Adjusted OR = 1.7, CI 0.2–0.603) were almost 1.8 times more likely to utilize outpatient services. Additionally, those with diabetes complications (OR = 1.93, CI 0.189–2.031) and those taking oral medication (OR = 3.131, CI 1.825–5.369) were respectively 1.9 and 3.1 times more likely to utilize inpatient care services.

**Conclusions:**

Our study showed that, although slum‐dwellers with type 2 diabetes needed outpatient services, a small percentage were referred to health centers and utilized health services. Multispectral cooperation is necessary for improving the status quo. There is a need to take appropriate interventions to strengthen health care utilization among residents with T2DM living in slum sites. Also, insurance organizations should cover more health expenditures and provide a more comprehensive benefits package for these patients.

## 1 BACKGROUND AND AIMS

A slum is an area that is characterized by deprivation and inequality.^1^ In the United Nations' Sustainable Development Goals (UN SDGs), slums are considered a global health priority.^2^ Several characteristics of slum‐dwelling can negatively affect slum residents' health, including insecure and poor‐quality houses, overcrowding, poor access to safe water, poor sanitation, and other infrastructure.^3^

Type 2 diabetes mellitus (T2DM), as one of the most common Non‐Communicable Diseases (NCDs), is an increasing public health problem worldwide.^4^, ^5^, ^6^, ^7^ Risk factors for developing diabetes include obesity, ethnic origin, social inequality, and the geographic distribution of diabetes, which vary in various countries, regions, or cities.^8^, ^9^ The number of people living with diabetes globally is projected to rise from about 537 million adults to 643 million and 784 million adults by 2030 and 2045. The large majority of these people will live in low‐ and middle‐income countries. According to International Diabetes Federation (IDF) report, over three in four adults with diabetes live in low‐ and middle‐income countries.^10^, ^11^ The economic burden of T2DM and its complications is substantial in these countries.^12^ Due to political, practical, cultural, and social issues, developing countries face difficulties in managing T2DM.^13^ Diabetes has become a crucial public health concern in Asian countries, particularly Iran.^14^, ^15^ According to the World Health Organization (WHO), 10% of Iran's population had diabetes in 2016.^14^, ^16^ The prevalence of diabetes is associated with poor living situations.^17^ The odds of inflicting diabetes are higher in poor neighborhoods.^18^ Therefore, disadvantaged groups such as slum dwellers are more prone to the prevalence, risk factors, and complications of T2DM.^19^ There are some behaviors and conditions that increase the risk of T2DM in slum dwellers. The weighted prevalence of behavioral factors that increase NCDs among slum‐dwellers in Kenya were unhealthy diet (57.2%), lack of physical activity (14.4%), tobacco use (12.4%), and alcohol abuse (10.1%).^20^ According to a study in Brazil, T2DM prevalence in slum‐dwellers was almost two‐fold higher than in the general population (10.1% vs. 5.2%).^21^ In addition, the disadvantaged groups are more likely not to adhere to standard health care.^22^ A study conducted in urban slums in India found that patients with type 2 diabetes had poor medication adherence.^23^ Under‐utilization of health care services is an adverse health outcome arising from slum‐dwelling.^3^ Despite unfavorable health status and environment and high‐level needs for health care, research demonstrated that those living in slum areas were less likely to seek and use health care services than those residing in the cities.^24^, ^25^ Slum residents have lower access to health care services for NCDs than their nonslum counterparts.^25^ A study in Iran showed that health services utilization among slum‐dwellers was not desirable.^26^ Results of a study in Tabriz found that medication underutilization due to high costs was higher in slum dwellers than in the general population (7/2% vs. 3/3%).^27^

To prevent the worsening of diabetes, and its complications, health care utilization in slum areas is highly important. To the best of our knowledge, there is insufficient study regarding health care utilization and its factors affecting slum dwellers in slum areas in Iran. To this end, this study aimed to examine the extent of health care utilization among slum‐dwellers with T2DM in Tabriz, Iran.

## 2 METHODS

### 2.1 Study design and setting

This cross‐sectional study was conducted among slum‐dwellers with T2DM in Tabriz, Iran, in 2022. Inclusion criteria were being over 18‐year‐old and living in slum areas for at least 5 years. We excluded those with other types of diabetes and those with mental disabilities. Our definition of health care utilization is the use of services and the number of services used.

Tabriz is a metropolis located in northwest Iran with almost two million inhabitants. According to municipal officials, the number of people living in slums in Tabriz has increased in recent years. At the time of the study, 3,82,124 people were living in slum areas, of which 13,155 had T2DM, as reported by the Vice‐Chancellor for Health of Tabriz University of Medical Sciences. In recent years, four health complexes have been established in slum areas of the city. Each health complex covers 3–4 health service centers. In general, there are 15 health service centers in slums.

### 2.2 Sample size and sampling method

According to Cochran's Sample Size Formula^28^ (*n* = N*t*2pq/Nd2 + t2pq), with a possible prevalence of 0.50, a confidence level of 95% and the marginal error of 0.05, the sample size was estimated at 374. We extracted the list of T2DM patients from the National Integrated Health System (SIB). Primary patients' characteristics including age, gender, phone/mobile number, and address are recorded at SIB. A code is assigned to each patient at SIB. We first stratified patients based on gender and 10‐year‐old groups. Then, nearly 100 patients were selected for each health complex using a systematic random sampling method. In this way, the population size was divided by the desired sample size. This fixed interval was used to select the patients. Finally, 400 patients were included and interviewed in our study.

### 2.3 Data collection tools

A researcher‐made questionnaire was used for data collection. To design the questionnaire, we identified potential needs and essential health care for patients with diabetes and the appropriate time intervals for them, according to Iran's Package of Essential Noncommunicable (IraPEN) disease. IraPEN is part of the National Health Transformation Plan (HTP), launched in 2014 by Iran's Ministry of Health and Medical Education (MoHEM), to provide universal health coverage, including access to NCDs prevention and care as well as mental health services.^29^ In 2014, the Health Transformation Program was launched to protect people from high medical costs, increase access to health care, and improve health care quality.^30^

The questionnaire encompasses two parts. The first part was related to demographic and socioeconomic variables such as age, gender, education status, marital status, income level, insurance coverage status, disease duration, treatment type, and complications (11 questions). The second part was related to questions on health services utilization (6 questions) (see Supporting Information: Appendix). Ten experts in the field assessed the questionnaire's face validity. They confirmed that the questionnaire covered the study's objectives. Data were collected via face‐to‐face interviews in a confidential setting within the health centers for 2 months. Those who were literate filled in the questionnaire. For those who were illiterate, the questions were read out to them by the two trained researchers and were asked to answer each question. We trained data collectors not to ask directional questions. As a result, we were relatively confident that the study was not biased. Because of the specific process of data collection and the low probability of data loss, our approach for missing data was to exclude cases pairwise.

### 2.4 Data analysis

Data were analyzed using SPSS version 22. Descriptive statistics (frequency and percent) were used to assess the extent of inpatient and outpatient services utilization. The *χ* ^2^ test was used to examine the association between sociodemographic variables and health care utilization. A Binary Logistic Regression model was applied to examine the effect of sociodemographic variables on health services utilization. A Generalized Linear Model was applied to examine the effect of variables on the frequency of health care utilization.

## 3 RESULTS

A total of 400 slum‐dwellers with T2DM were included in our study. More than half of the participants were female (53.8), and most of them were below the age of 60 years. Ninety percent of participants were married, and almost 60% of them were illiterate or were able to read and write only. The majority of respondents (75.8%) had an income level of 1 < 40 million Rial. The majority of patients (82.3%) were covered by basic health insurance, and only 15% were covered by supplemental insurance. Moreover, the disease duration of most participants was >5 or 5–10 years. Approximately 60% of subjects took oral medication, and more than 70% had complications (Table 1).

**Table 1:** Demographic and socioeconomic characteristics of the study population and its association with service utilization.

Variables	Categories	Frequency (percent)	Outpatient services			Inpatient care		
			Utilization %	*χ* ^2^	*p* Value	Utilization %	*χ* ^2^	*p* Value
Gender	Male	185 (46.2)	34.1	2.56	0.10	38.9	0.12	0.72
	Female	215 (53.8)	41.9			37.2		
Age (in years)	Under 30	38 (9.5)	31.6	1.154	0.886	7.9	44.586	<0.0001
	30–40	91 (22.8)	37.4			29.7		
	40–50	82 (20.5)	37.8			29.3		
	50–60	87 (21.8)	41.4			40.2		
	Over 60	102 (25.5)	39.2			61.8		
Education	Illiterate	111 (27.8)	38.7	1.885	0.597	50.5	33.982	<0.0001
	Reading and writing ability	121 (30.3)	39.7			48.8		
	Diploma	131 (32.8)	34.4			25.2		
	Academic education	37 (9.3)	45.9			10.8		
Marital status	Single	40 (10.0)	43.5	1.383	0.240	42.4	0.978	0.323
	Married	308 (90.0)	36.7			36.7		
Income (The Iranian Rial)	<40 million	303 (75.8)	35.3	4.562	0.033	39.9	1.984	0.159
	40–80 million	78 (24.3)	47.4			32		
Basic insurance	Yes	329 (82.3)	41.0	6.080	0.014	38.3	0.070	0.792
	No	71 (17.8)	25.4			36.6		
Type of Insurance	Social insurance	193 (57.8)	47.2	10.433	0.005	37.8	2.894	0.235
	Iran health insurance	114 (34.1)	28.9			34.2		
	Others	27 (8.1)	48.1			51.9		
Supplemental Insurance	Yes	60 (15.0)	51.7	5.380	0.020	36.7	.053	0.817
	No	340 (85.0)	35.9			38.2		
Disease duration (years)	>5	114 (28.5)	35.1	5.564	0.234	19.3	30.475	<0.0001
	5–10	104 (26.0)	33.7			38.5		
	10–15	55 (13.8)	43.6			34.5		
	15–20	40 (10.0)	52.5			57.5		
	<20	46 (11.5)	37.0			56.5		
Treatment type	Lifestyle change	27 (6.8)	44.4	16.806	0.001	25.9	43.594	<0.0001
	Oral pills	236 (59.0)	30.5			26.7		
	Insulin	17 (4.3)	35.3			47.1		
	Mixed regime simultaneously	120 (30.0)	52.5			61.7		
Diabetes complications	Yes	286 (71.5)	45.1	19.964	<0.0001	51.0	72.527	<0.0001
	No	114 (28.5)	21.1			5.3		

As shown in Table 1 outpatient services utilization was significantly associated with income level (*p* < 0.0001), basic insurance (*p* = 0.014), type of insurance, supplemental insurance (*p* = 0.02), treatment type (*p* = 0.001), and diabetes complications (*p* < 0.0001). Those with higher income levels, who were covered by social and supplemental insurance, those who modified their lifestyle, and those with diabetes complications were more likely to utilize outpatient services. We also found that age (*p* < 0.0001), education level (*p* < 0.0001), disease duration (*p* = 0.0001), treatment type (*p* < 0.0001), and diabetes complications (*p* < 0.0001) were significantly associated with inpatient care utilization. Older and illiterate patients, those with disease duration between 5 and 10 years, those with mixed regime simultaneously, and those with diabetes complications were more likely to utilize inpatient care.

**Figure 1:**
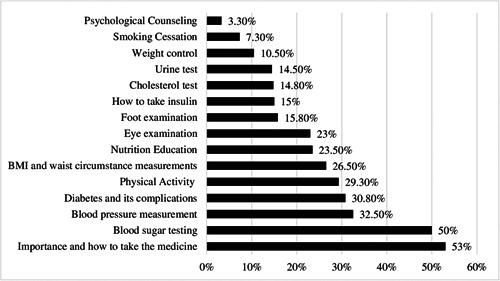
Percentage of patients receiving health care services during the past 3 months.

Figure 1 shows the percentage of patients with T2DM referring to health centers and utilizing essential health care services. The most frequently utilized services among patients were the importance and how to take medicine (53%), blood sugar testing (50%), and blood pressure measurement (32.50%). The least frequently utilized health services were psychological counseling (3.30%), smoking cessation (7.30%), and weight control (10.50%).

**Figure 2:**
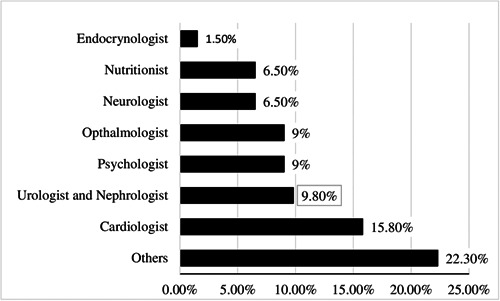
The percentage of specialists that patients refer to for receiving services.

As shown in Figure 2, patients with diabetes complications were mostly referred to cardiologists (15.80%), urologists, nephrologists (9.80%), or other specialists (22.30%) during the last year.

The relative frequency of outpatient services and inpatient care utilization were 0.76 and 0.96, respectively. The results showed that although 49.8% of patients needed outpatient services, only 38.3% were referred to health centers and utilized health services during the past 4 weeks. Additionally, 38% out of 39.5% of patients who needed inpatient care services, were referred to health centers and utilized these services in the last year (Table 2).

**Table 2:** Utilization of outpatient services and inpatient care.

Variables	Categories	Outpatient services	Inpatient care
		Frequency (percent)	Frequency (percent)
		During the past 4 weeks	During the last year
Feeling a need	Yes	199 (49.8)	158 (39.5)
	No	201 (50.2)	242 (60.5)
Utilization	yes	153 (38.3)	152 (38.0)
	No	247 (61.7)	248 (62.0)
Frequency of use	0	247 (61.7)	248 (62.0)
	1	99 (24.8)	74 (18.5)
	2	40 (10)	44 (11.0)
	3	14 (3.5)	18 (4.5)
	4	0	16 (4)

We applied a binary logistic regression model for the dependent variables (utilization = 0, nonutilization = 1). Based on Hosmer and Lemeshow test, the logistic model was suitable (*χ* ^2^ = 4.381, *p* = 0.0821). The results showed women (OR = 1.871, *p* = 0.009), those with higher income levels (OR = 1.984, *p* = 0.022), those taking oral medication (OR = 1.982, *p* = 0.012), and those with diabetes complications (OR = 1.7, *p* < 0.001) were almost 1.8 times more likely to utilize outpatient services. Additionally, those with diabetes complications (OR = 1.93, *p* < 0.001) and those taking oral medication (OR = 3.131, *p* < 0.001) were respectively 1.9 and 3.1 times more likely to utilize inpatient care services. To summarize, patients with diabetes complications and those taking oral medication were more likely to utilize outpatient and inpatient care services (Table 3).

**Table 3:** **Binary logistic regression model for factors affecting utilization**.

Variables	Outpatient services				Inpatient care			
	*p* Value	Adjusted OR	95% CI for OR adjusted		*p* Value	Adjusted OR	95% CI for OR adjusted	
			Lower	Upper			Lower	Upper
*Gender (reference = male)*								
Female	0.009*	1.871	1.170	2.993				
*Income (reference > 4 million)*								
4–8 million	0.022*	1.984	1.105	3.562				
*Treatment type (reference = mixed regime simultaneously)*								
Lifestyle change	0.912	1.058	0.391	2.858	0.518	1.461	0.462	4.618
Oral medication	0.012*	1.982	1.166	3.369	<0.001*	3.131	1.825	5.369
Insulin	0.737	1.248	0.342	4.559	0.915	0.932	0.256	3.391
*Complications (reference = no)*								
Yes	<0.001*	1.7	0.200	0.603	<0.001*	1.93	0.189	2.031

* Significant was considered for *p* < 0.05

we used a generalized linear model to predict variables that influence the number of outpatient and inpatient utilization. Table 4 shows the results of the generalized linear model. Those with diabetes complications, especially those with comorbidities, were more likely to utilize outpatient services (*p* < 0.001). Additionally, those taking oral medication (*p* < 0.001) and those with diabetes complications (*p* < 0.001) were more likely to utilize inpatient care services (Table 4).

**Table 4:** Generalized linear model for factors affecting the number of health care utilization.

Variables	Outpatient services				Inpatient care			
	*p* Value	Wald *χ* ^2^	95% CI for Wald *χ* ^2^		*p* Value	Wald *χ* ^2^	95% CI for Wald *χ* ^2^	
			Lower	Upper			Lower	Upper
*Treatment type (reference = mixed regime simultaneously)*								
Lifestyle change					0.148	2.093	−0.817	0.123
Oral pills					<0.001*	18.138	−0.798	−0.295
Insulin					0.313	1.018	−0.918	0.294
*Comorbidities (reference = no)*								
Yes	<0.001*	15.571	0.192	0.571	<0.001*	17.473	0.300	0.831

* Significant was considered for *p* < 0.05

## 4 DISCUSSION

This study aimed to assess the extent of health care utilization among slum‐dwellers with T2DM in Tabriz, Iran. Our results showed that although slum‐dwellers with T2DM needed outpatient services, only a small percentage were referred to health centers and utilized health services. A study in Iran showed that 45% of those with T2DM forgone treatment because of financial constraints and dissatisfaction with the quality of treatment.^31^ Although one of the goals of IraPEN was higher access to care for NCDs, including T2DM,^32^, ^33^ utilization of essential health care among slum‐dwellers was considerably low. According to this plan, patients with diabetes should receive essential health care services once every 3 months. In our study, 50%, 85%, and 68% of slum‐dwellers, respectively, reported not being referring health centers to utilize blood sugar tests, cholesterol tests, and blood pressure measurements during the past 3 months. T2DM, blood pressure, and high cholesterol are the most common risk factors for developing diabetes complications such as retinopathy and neuropathy.^34^ Notably, in our study, patients with T2DM reported they mostly visited cardiologists, urologists, and nephrologists. It seems that diabetes‐related complications are high among these groups. Due to their low socioeconomic status, they may avoid visiting General Practitioners (GPs), specialists, and health care centers regularly.

In the current study, almost 97% of participants reported that they did not utilize psychological counseling. In line with our results a systematic review and meta‐analysis study showed that nearly 97% of patients with diabetes did not receive psychological counseling, while patients with diabetes are more at risk to psychological complications.^35^ This may be due to sociocultural barriers in these communities deterring them from visiting psychologists and receiving psychological counseling. Studies reported that depression and diabetes distress are common in people with T2DM, especially in older adults. This impedes them from controlling disease and reduces control of HbA1c, blood pressure, and cholesterol, treatment adherence, and overall health outcomes.^35^, ^36^ Thus, patients with diabetes require access to mental health services in addition to governing metabolic abnormalities.^37^ Health care providers should promote self‐care management education and support these patients, as self‐management education can reduce psychological factors such as depression and distress and improve diabetes control and the overall quality of life in people with T2DM.^38^

Our results confirmed the results of a study that uninsured patients with type 2 diabetes are less likely to utilize health care services.^39^ In our study, patients with social and supplemental insurance coverage used more health care services when it came to outpatient services. A probable explanation is that social insurance mostly covers employees, so patients with this type of insurance enjoy better economic and social status. Moreover, supplemental insurance covers costs and services not covered by basic health insurance^40^ and reduces financial barriers. However, seeking supplemental insurance is probably related to higher income.^41^ A strategy to increase access to health services and protect members from the financial risks of diseases for poor people, especially informal sector workers, is community‐based health insurance schemes.^42^, ^43^ Study findings in Ethiopia indicate that households with community‐based health insurance utilize health care more than households without insurance (50.5% vs. 29.3%).^44^

Although income was a determinant factor in outpatient service utilization, most patients in our study had a low‐income level. A small increase in income level can lead to an increase in outpatient services utilization. This result is supported by previous studies that the leading cause of forgoing care among T2DM was financial constraints.^40^ On the contrary, our results did not show a significant association between income level and inpatient care utilization. This might be due to some reasons. Inpatient services are more inelastic, and patients have to use them for survival. Additionally, insurance's copayment for inpatient services is <10% versus 30%–50% for outpatient services.

We also found that patients with a university education were more likely to use outpatient services, while those with low education were more likely to use inpatient care. Liu and colleagues showed that low education levels was correlated with poorer socioeconomic circumstances and increased risk for T2DM.^45^ It is not difficult to understand that those with higher education have more knowledge of the disease, have better health‐seeking behaviors, and consequently are more likely to practice self‐care behaviors. Self‐care knowledge can influence health care utilization,^46^ and health literacy leads to better diabetes management.^47^ In addition, patients with lower education levels may lack enough knowledge of the disease and postpone their treatment process. As a result, due to disease progression, they are more likely to be hospitalized in health centers and utilize inpatient care services. The inpatient care utilization in this study was higher among older adults. As patients age, they may use these services more. Statistics indicated that after South Korea, Iran is the second‐fastest ageing country in the world.^48^ The prevalence of diabetes and its complications may increase with age. Thus, this calls for more resources and manpower for managing and controlling the disease.

In the current study, patients with longer disease duration were more likely to use inpatient care services. Patients with diabetes may experience complications, such as retinopathy, leading to longer diabetes duration.^49^ According to our findings, taking oral medication can increase the frequency and likelihood of utilizing outpatient and inpatient care services.

### 4.1 Strengths and limitations

Our study has strengths and limitations that deserve to be mentioned. Its main strength is that this study was conducted among those who have been marginalized. Moreover, The results of the study provide a relatively comprehensive picture of socioeconomic factors influencing health care utilization among slum‐dwellers. With respect to limitations, no comparison was conducted between those residing in slums and those residing in urban areas regarding the extent of health care utilization. The study population was selected from only one city. This may affect the generalizability of the results. Finally, we didn't assess all factors influencing health care utilization, including lifestyle and behavior.

### 4.2 Policy implications

Our study shed light on the problems of utilizing health services among patients with T2DM in slum areas. First, a small percentage of patients were referred to health centers and utilized outpatient services, although most of them left feeling a need. It seems IraPEN goals to improve access to health centers are unmet. Decreasing medical prices, rising income levels, and implementing health insurance can increase health service utilization. Also, to increase health service utilization, a holistic approach should give attention to the demand and supply side of health care delivery. Second, older and illiterate patients utilize more inpatient care services, so appropriate educational interventions and strategies such as self‐care management education are needed to enhance patients' knowledge of the disease. Third, those with social and supplemental insurance were more likely to use outpatient and inpatient services. Therefore, the government should provide proper financial provisions for underprivileged patients and increase the reimbursement of treatment through medical insurance and supplemental insurance coverage. Finally, we recommend Community‐based health insurance schemes to improve health care utilization among slum‐dwellers with T2DM.

## 5 CONCLUSIONS

Our study showed that, although slum‐dwellers with type 2 diabetes needed outpatient services, a small percentage were referred to health centers and utilized health services. Older and illiterate patients, those with higher income levels, with social and supplemental insurance coverage, those who modified lifestyles, those with diabetes complications, and those with longer disease duration were more likely to utilize outpatient and inpatient care services. Using the results of the analysis, options are suggested to improve the status quo. The government should pay special attention to slums due to their sociodemographic characteristics. Because of low education in slums, the health system must provide adequate education about diabetes and the importance of its control. Government should support slum‐dwellers with T2DM through subsidies, so they could be covered by supplementary insurance for the neighbors. Iran's law on public insurance coverage needs to be implemented. Iran's health insurance organization should consider sufficient coverage of costs and expand the benefit package for slum‐dwellers. Finally, we recommend a qualitative study among those with T2DM for a deeper understanding of barriers to health care utilization.

## AUTHOR CONTRIBUTIONS

**Fawzieh Ghammari**: Conceptualization; methodology; writing—original draft. **Habib Jalilian**: Formal analysis; writing—original draft. **Rahim Khodayari‐zarnaq**: Writing—review & editing. **Masumeh Gholizadeh**: Methodology; supervision; writing—review & editing.

## CONFLICT OF INTEREST STATEMENT

The authors declare no conflict of interest.

## ETHICS STATEMENT

This study was approved by the Ethics Committee of the Tabriz University of Medical Science (Reference No: IR.TBZMED.REC.1400.961). Informed written consent was obtained from participants. Researchers read out an informed consent form for illiterate patients and obtained informed written consent from their legal guardians. Participants were assured that their personal information would remain confidential.

## TRANSPARENCY STATEMENT

The lead author Masumeh Gholizadeh affirms that this manuscript is an honest, accurate, and transparent account of the study being reported; that no important aspects of the study have been omitted; and that any discrepancies from the study as planned (and, if relevant, registered) have been explained.

## Supporting information

Supporting information.

## Data Availability

The data that support the findings of this study are available from the corresponding author upon reasonable request.
