# Supplementary material for: Health care utilization and its association with sociodemographic factors among slum‐dwellers with type 2 diabetes in Tabriz, Iran: A cross‐sectional study
Source: Health Sci Rep. 2023 May 25;6(5):e1272. doi: 10.1002/hsr2.1272 (PMC10213483; doi:10.1002/hsr2.1272)
Supplement: Supplementary file 1 — Supporting information. [file HSR2-6-e1272-s001.docx]

**Dear participant**

This questionnaire is designed to investigate the health care utilization in slum-dwellers with type 2 diabetes. Your participation in this research can help improve health care utilization to manage your disease, so please answer the questions carefully. It should be noted that your participation in this research is completely voluntary. Your name and personal information will be kept anonymous.

Thank you in advance for your cooperation.

Email: [fgh12@ymail.com](mailto:fgh12@ymail.com)

- **Socio-demographic questions**

1. **Gender**

Male 🞎 Female 🞎

1. **Age**

>30 🞎 30-40 🞎 40-50 🞎 50-60 🞎 <60 🞎

1. **Marital status**

Single 🞎 Married 🞎

1. **Education**

Illiterate 🞎 Reading and writing ability 🞎 Diploma 🞎 University education 🞎

1. **Income**

<40 million🞎 40-80 million 🞎 >80 million 🞎

1. **Are you covered by any basic health insurance?**

Yes 🞎 No 🞎

1. **Type of Insurance**

Social insurance 🞎 Iranian health insurance 🞎 others 🞎

1. **Are you covered by any supplemental health insurance?**

Yes 🞎 No 🞎

1. **Disease duration (in years)**

>5 🞎 5-10 🞎 10-15 🞎 15-20 🞎 >20 🞎

1. **Treatment type**

Lifestyle change 🞎 Oral pills 🞎 Insulin 🞎 Mixed regime simultaneously 🞎

1. **Diabetes complications**

Yes 🞎 No 🞎

- **Health care utilization questions**

**12. Which services or consultations have you received during the past three months?**

| **Type of service** | **Yes** | **No** |
| --- | --- | --- |
| Cholesterol test |  |  |
| BMI and waist measurements |  |  |
| Blood sugar testing |  |  |
| Eye examination |  |  |
| Blood pressure measurement |  |  |
| Foot examination |  |  |
| Urine test |  |  |
| Cholesterol test |  |  |
| Diabetes complications |  |  |
| Importance and how to take the medicine |  |  |
| How to take insulin |  |  |
| Weight control |  |  |
| Nutrition |  |  |
| Quitting smoking |  |  |
| Physical activity |  |  |
| Psychological counseling |  |  |

**13. Have you felt the need to visit a medical specialist due to complications of diabetes during the last year?**

Yes 🞎 No 🞎

**13-1. If your answer is yes,** which medical specialist have you visited?

Endocrinologist 🞎

Cardiologist 🞎

Urologist and nephrologist 🞎

Psychologist 🞎

Ophthalmologist 🞎

Neurologist 🞎

Nutritionist 🞎

Others 🞎

**14- Have you needed outpatient services in the past four weeks?**

Yes 🞎 No 🞎

**14-1. If your answer is yes**, you were able to receive it.

Yes 🞎 No 🞎

**15- How many times have you received outpatient service during the past four weeks?**

0 🞎 1 🞎 2 🞎 3 🞎 4 🞎

**16. Have you needed inpatient care during the last year?**

Yes 🞎 No 🞎

**16-1. If your answer is yes, you were able to receive it.**

Yes 🞎 No 🞎

**17. How many times have you received inpatient care in the last year?**

0 🞎 1 🞎 2 🞎 3 🞎 4 🞎
